# Measuring the Compression Force Required for Vascular Shortening in Ultrasonic Vascular Models

**DOI:** 10.7759/cureus.32596

**Published:** 2022-12-16

**Authors:** Nao Sato, Kenji Karino

**Affiliations:** 1 Clinical Skill Up Center, Shimane University Hospital, Shimane, JPN

**Keywords:** ultrasonic vascular models, compression force, ultrasound, deep vein thrombosis, ultrasonography

## Abstract

Background

Vascular compression is important for deep vein thrombosis screening. However, pressure analysis of ultrasound vessel models has not been performed. Therefore, we compared the human popliteal vein and several ultrasound vessel models at 50% compression.

Methodology

Four major ultrasound vascular models used in Japan and the popliteal vein of one subject constituted our measurement targets. Using a pressure-sensitive measuring device, the compressive force required to shorten the vessel diameter by 50% was determined.

Results

The compression force that shortened the popliteal vein by 50% was measured to be 191 ± 65 g. The blue phantom, ultrasound CV Pad II, ultrasound training block, and UGP-GEL required compression force of 701 ± 8 g, 265 ± 12 g, 697 ± 20 g, and 745 ± 15 g, respectively. The compression force for the ultrasound training block was 2.6 times higher than that for the ultrasound CV Pad II. The gel material around the vessels was the same; however, different vascular tubes required 2.6 times higher compression force.

Conclusions

This study showed that the objective numerical values of the compressive force were required to compress an ultrasound vascular model. Reproduction of the compressibility of veins required either removing the vascular structure or using thin tubing material.

## Introduction

Thrombi can form because of a variety of factors, with the most common being stagnation of blood flow, damage to the endothelium, and increased blood clotting. These three factors are referred to as Virchow’s triad and are considered risk factors for thrombosis [1]. Overlapping of these factors and a high risk of developing deep vein thrombosis (DVT) is considered serious. People often spend most of their time with their legs lowered or flexed because of the lack of adequate living space in shelters and cars, which can lead to stagnation of blood flow. Bruising and trauma can also damage the vascular endothelium. Insufficient water intake, suppression of fluid intake, and sympathetic dominance due to stress can increase the viscosity of blood and promote coagulation.

Screening conducted in evacuation centers after the Kumamoto earthquake showed that 10% of disaster victims had DVT [2]. In addition, the prevalence of DVT in evacuation centers inundated by tsunamis during the great East Japan earthquake was 200 times higher than that previously reported in Japan [3]. Compared to evacuation centers that were not inundated by the tsunami, inundated centers faced problems such as deteriorating sanitary conditions owing to the influx of sludge, delays in relief supplies due to the severing of roads, and continued congestion in evacuation centers due to the large number of people having difficulty returning home. The area occupied per victim was approximately one tatami mat for at least one month [4]. In Japan, where disasters are frequent, early detection of DVT and prevention of pulmonary thromboembolism and other diseases are important. Therefore, high-quality training of ultrasound technicians is urgently required.

To train ultrasound technologists, increasing examination opportunities is important. However, it is undesirable to examine real patients without sufficient training. Therefore, we gathered those who were willing to cooperate as simulated patients for training technologists in ultrasound examinations on a daily basis. However, it takes several hours to train several learners in the technique. In addition, a few simulated patients find examining the femoral vein embarrassing because it requires exposure of the groin. Thus, it is difficult to always obtain the cooperation of a simulated patient, and the use of a vascular model or simulator is effective. Training using ultrasound phantoms is an effective way to improve ultrasound skills [5,6].

For example, we use the central venous model and the peripherally inserted central venous model to train catheter insertion. Various other ultrasound phantoms are also used for education, such as lung ultrasound training phantom and rheumatoid hand phantom with synovial fluid retention [7,8]. However, there are currently no ultrasound training phantoms to train for DVT detection. Therefore, various homemade vascular models using three-dimensional (3D) printers, tofu, gelatin bases, etc. have been reported, but they are not widely used [9-13]. We utilize ultrasound practice, ultrasound training block, and central venous models for vein delineation practice, but the thickness and brightness of the vessels vary from model to model. Ultrasound training blocks and blue phantom are vessel models that do not distinguish between arteries or veins, and they also differ in stiffness when compressed [14-17]. Vein compression is an important observation point in DVT screening [18]. To date, no studies have compared the force required for vascular compression in different vascular models. This study investigated the pressure required for vascular compression in an ultrasound vascular model and examined the reproduction of veins using the compression method.

## Materials and methods

Ethical approval

The purpose and methods of the study were disclosed and participants were recruited. One subject who agreed to participate in the study underwent measurement of the popliteal vein. We verbally explained that only age, gender, and body mass index (BMI) would be disclosed and that we would not identify any individual. Consent was obtained after explaining that the results obtained in this study would be published in medical societies and medical journals. As this work involved the development of an educational tool, approval from the Institutional Review Board was not required.

Measurement object

**Figure 1:**
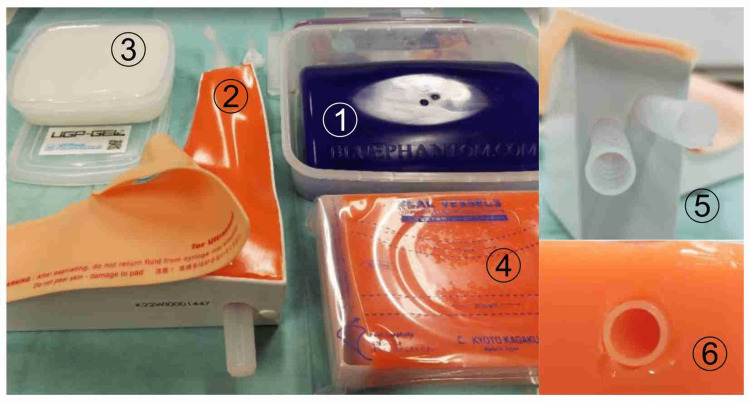
Ultrasound vessel models. (1) Blue phantom (CAE Healthcare, USA). (2) Ultrasound CV Pad II (Kyoto Kagaku, Japan). (3) Real Vessel (Kyoto Kagaku, Japan). (4) UGP-GEL (ALFABIO, Japan). (5) Ultrasound CV Pad II soft tube that reproduces blood vessels. (6) Rigid tubing that replicates real vessels.

The following four major vessel models used in Japan were employed: blue phantom (CAE Healthcare, USA), ultrasound CV Pad II (Kyoto Kagaku, Japan), ultrasound training block (Kyoto Kagaku, Japan), and UGP-GEL (ALFABIO, Japan) (Figure 1). The ultrasound CV Pad II and ultrasound training block were made of a urethane resin, and UGP-GEL was made of agar. The blue phantom and UGP-GEL did not have vascular tubes; the holes were filled with mucus. We measured the popliteal vein of one subject to compare it with that of a human subject. The subject was a male in his 30s with a BMI of 22 kg/m^2^.

Ultrasonic equipment and pressure-sensitive measurement system

The ultrasound system, Xario SSA-660A (Toshiba Inc., Japan), with a linear probe (7 MHz) weighing 200 g was used. The pressure required to compress the vessel was measured using a pressure-sensitive sensor and pressure-sensitive measurement system (Canon Chemicals Inc., Japan), both of which were employed to measure the pressure required for vascular compression. The pressure-sensitive sensor can measure pressures in the range of 2-50 kg. This study used a micro-pressure sensor (CKS18L-F, Ф18 mm, thickness 0.17 mm). The sampling rate of the pressure-sensitive measurement system was 0.02 seconds.

Pressure measurement method

**Figure 2:**
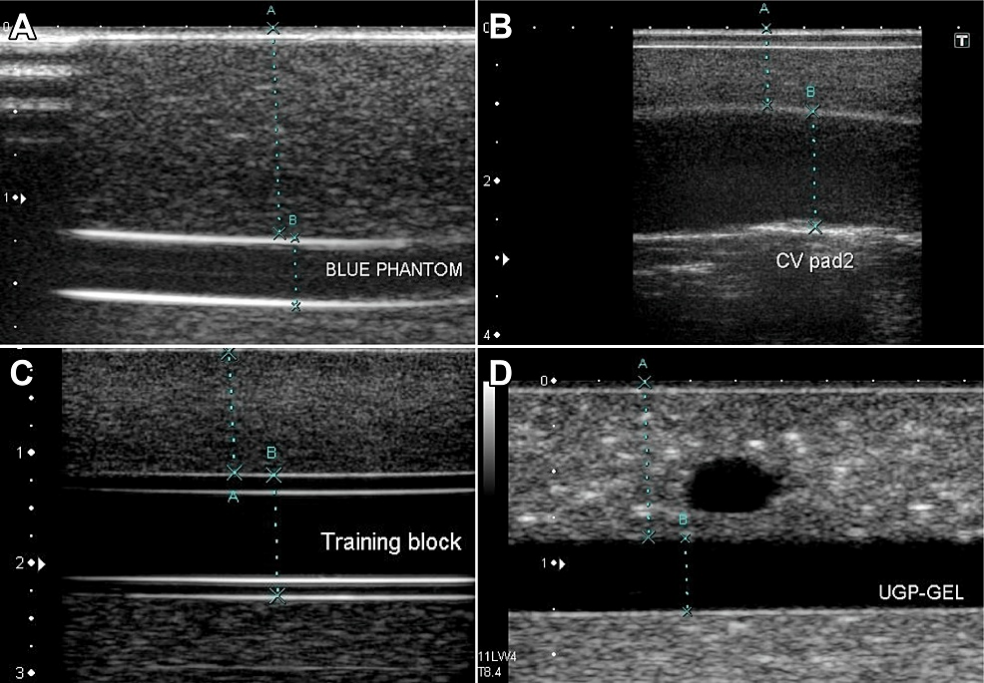
Vessel models in the long-axis image. Long-axis view of (A) blue phantom, (B) ultrasound CV Pad II, (C) real vessel, and (D) UGP-GEL.

A pressure-sensitive sensor was placed on the blood vessel model, and the probe was applied on top of the sensor for measurement. We drew a long-axis image to visualize the structure of the vessel model (Figure 2). The diameter, pressure, and depth of the vessels were measured without applied pressure. The pressure was continuously measured by applying the probe for 10 seconds. The 10-second average (500 samples) was considered the representative value and was compared with four different vessel models and the popliteal vein. Subsequently, the pressure during compression was measured.

Statistical analysis

The data obtained from pressure-sensitive measurements were tested for equal variances using the F-test after confirming normality. This was followed by Welch’s t-test. JMP Pro 16.2.0 (SAS Institute, Cary, NC, USA) was used for statistical analysis. In all tests, a p-value of 0.05 was considered statistically significant.

## Results

Vascular depth and diameter

The vessel depth and diameter of the popliteal vein were 11.5 mm and 5.0 mm, respectively; those of blue phantom were 12.0 mm and 5.0 mm, respectively; those of ultrasound CV Pad II were 10.0 mm and 15.0 mm, respectively; those of ultrasound training block were 11.0 mm and 11.0 mm, respectively; and those of UGP-GEL were 8.5 mm and 4.0 mm, respectively (Table 1).

**Table 1:** Vascular depth and diameter.

	Vessel depth (mm)	Vessel diameter (mm)	Pressure to shorten blood vessels by 50 (%)
Popliteal vein	11.5	5.0	191 ± 65
Blue phantom	12.0	5.0	701 ± 8
CV Pad II	10.0	15.0	265 ± 12
Real vessel	11.0	11.0	697 ± 20
UGP-GEL	8.5	4.0	745 ± 15

Probe weight loaded on the vascular model

The probe weights for long-axis image rendering were measured. The weights loaded on the blue phantom, ultrasound CV Pad II, ultrasound training block, and UGP-GEL were 44 ± 2 g, 50 ± 4 g, 53 ± 4 g, and 57 ± 13 g, respectively.

Pressure to shorten blood vessels by 50%

**Figure 3:**
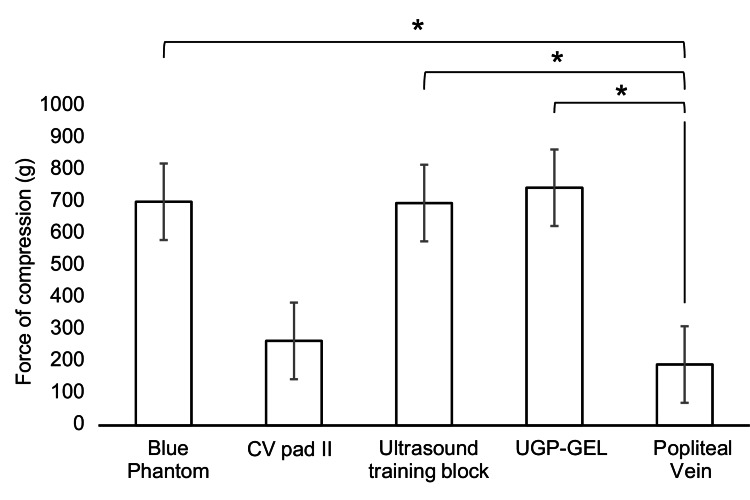
Compression force that shortens blood vessels by 50%. *: P < 0.05 indicates a significant difference between models.

Significant pressure differences were found in the vascular models except for ultrasound CV Pad II compared to the popliteal vein. The compression shortening the popliteal vein and each vascular model by 50% between blue phantom and popliteal vein (701 g, 95% confidence interval (CI) (698-702) and 191 g, 95% CI (185-196); p < 0.05), ultrasound CV Pad II and popliteal vein (265 g, 95% CI (263-265) and 191 g, 95% CI (185-196); p = 0.96), ultrasound training block and popliteal vein (697 g, 95% CI (692-698) and 191 g, 95% CI (185-196); p < 0.05), and UGP-GEL and popliteal vein (745 g, 95% CI (741-746) and 191 g, 95% CI (185-196); p < 0.05) were compared (Figure 3).

## Discussion

In this study, we compared the compression force required to compress the vascular model. The results revealed that the ultrasound CV Pad II was the closest to the human body in terms of the force required to shorten the vessel by 50%. Proximal compression ultrasonography, which observes two points, the common vena cava and the popliteal vein, is used to evaluate DVT [18-20]. This method is used as a point-of-care ultrasound examination in disaster and emergency medicine owing to its ability to reduce examination time. Mastery of compression techniques is an important issue. Various vascular models are available for the acquisition of ultrasound techniques. However, they are expensive and cannot be fully utilized in educational settings. To solve these problems, self-made ultrasound vascular models have been reported. For example, a high-quality vascular model that reproduces blood flow in leg veins using a micro-pump has been reported [11]. In addition, as an innovative model that pursues simplicity, a vascular model fabricated with different gelatin concentrations has been reported [10]. Thus, ultrasound-based vascular models have been developed from multiple perspectives, but to our knowledge, there are no vascular models focusing on the reproduction of compressive forces. Excessive compression underestimates the presence of DVT and risks releasing a thrombus. Therefore, the development of a vascular model that can disappear veins with appropriate pressure is required. To develop a pressure-specific vascular model, validating the characteristics of existing vascular models is necessary.

The ultrasound CV Pad II and ultrasound training block were manufactured by the same company, and the gel material around the blood vessel was the same in both cases. However, the force required for vascular compression was 2.6 times greater than that required for CV Pad II (approximately 430 g). The effect of depth was considered to be small because the difference in depth to the blood vessel was 1 mm. Therefore, we believe that vascular structure has the greatest influence on reproducing venous compression. The ultrasound training block uses thick silicone tubing, preventing pressure transmission and depicting an artery-like vessel wall. However, the tube of the ultrasound CV Pad II was thin and soft. Therefore, to reproduce veins, using tubing made of thin material is recommended.

This study has three limitations. The subject for comparison with multiple vascular models was a single adult male with a BMI of 22 kg/m^2^. Further studies with subjects of various demographics are required in the future. Second, assuming pressure attenuation due to differences in gel material, we compared the weight of the probe loaded on each vessel model and found no difference. This result does not exclude the possibility that the examiner unconsciously adjusts the pressure according to the softness of the vascular model. In the future, it is recommended that the probe be fixed to the machine for measurement. Third, we did not consider the role of model vein diameter on force distribution. The vessel diameters of the ultrasound CV Pad II and ultrasound training block used in this study were approximately three times larger than those of human popliteal veins. Further research is needed to determine how vessel diameter affects force distribution.

## Conclusions

Reliable vascular training models are required to train ultrasound examiners on lower extremity veins. In this study, we compared several ultrasound vascular models to the human popliteal vein at a 50% compression rate. The results revealed that the vascular model that most closely resembles the human popliteal vein uses thin tubing. Although the material used to reproduce fat and muscle is important, we found that the tubing material used to reproduce the blood vessels has an impact on the reproduction of compression.
